# A Paradox of Ethics: Why People in Good Organizations do Bad Things

**DOI:** 10.1007/s10551-022-05142-w

**Published:** 2022-05-27

**Authors:** Muel Kaptein

**Affiliations:** RSM Erasmus University Rotterdam, Room T11-53, P.O. Box 1730, 3000 DR Rotterdam, The Netherlands

**Keywords:** Unethical behavior, Good barrel approach, Threatening forces

## Abstract

This article takes a novel approach to explaining the causes of unethical behavior in organizations. Instead of explaining the unethical behavior of employees in terms of their bad organization, this article examines how a good organization can lead to employees’ unethical behavior. The main idea is that the more ethical an organization becomes, the higher, in some respects, is the likelihood of unethical behavior. This is due to four threatening forces that become stronger when an organization becomes more ethical. These forces are the upward, downward, backward, and forward forces. Each of these forces is illustrated with two effects and each effect is explained by a specific theory. The effects are the effects of the gold digger, high-jump bar, retreating-cat, forbidden-fruit, cheese slicer, moving-spotlight, repeat-prescription, and keeping-up appearances. This paradox of ethics, when goodness breeds badness, opens new research directions.

## Introduction

Unethical behavior is observed frequently in organizations. Research by Ethics and Compliance Initiative (2021) shows that 49% of U.S. employees reported observing unethical behavior in their organization. The most frequently observed types of unethical behavior were favoritism toward certain employees (35%), management lying to employees (25%), conflicts of interest (23%), improper hiring practices (22%), abusive behavior (22%), and health violations (22%). The research by Ethics & Compliance Initiative also shows that the rate of observed unethical behavior by U.S. employees has remained relatively steady over the past 20 years. Research in many other countries also shows that employees observe unethical behavior frequently in organizations (Ethics & Compliance Initiative, 2021; Institute for Business Ethics, 2021).

A popular approach for explaining unethical behavior in organizations is the bad barrel approach. This approach holds that unethical behavior in organizations is not only explained by the “bad apples”—the bad employees in an organization—but also by the “bad barrel”— the bad organization—itself (Ashkanasy et al., 2006; Kish-Gephart et al., 2010; Treviño & Youngblood, 1990). The organization is bad, i.e., unethical, when aspects of the organizational context, like the organization’s ethical climate and ethical culture, stimulate employees to behave unethically (Kish-Gephart et al., 2010). The bad barrel approach requires an understanding of those factors that make the organization bad (Treviño et al., 2014).

This article takes a novel approach to explain the causes of unethical behavior in organizations; we call it the *good* barrel approach. As the name suggests, instead of explaining unethical behavior in terms of the badness of the organization, this approach explains unethical behavior in terms of the goodness of the organization. Using this approach raises the question regarding which factors inside and outside the organization are activated and strengthened when an organization becomes better, i.e., more ethical, relative to how it was before. The good barrel approach helps identify specific factors that stimulate unethical behavior and that are related to the goodness, but not to the badness, of an organization. Identifying these specific factors would explain why it is increasingly difficult for organizations to become and stay ethically good. To understand what it means when an organization becomes more ethical, this article uses Reidenbach and Robin’s (1991) model of the corporate moral development. To understand the factors that emerge and intensify when an organization develops, the literature on organizational life cycles is used. These factors, which are composed of forces and their effects, are suggested to become stronger, i.e., more influential, when an organization becomes more ethical.

This article identifies four threatening forces and illustrates each with two effects. The four forces are the upward, downward, backward, and forward forces. The upward force is illustrated with the gold digger and high-jump bar effects, the downward force with the retreating-cat and forbidden-fruit effects, the backward force with the cheese slicer and moving-spotlight effects, and the forward force with the repeat-prescription and keeping-up appearances effect. Each effect is explained by a specific theory; these are, respectively, the social identity theory (Albert & Whetten, 1985), theory of moral progress (Kaptein, 2021), organizational trust theory (Mayer et al., 1995), forbidden-fruit theory (Bushman & Stack, 1996), resource dependence theory (Durand et al., 2019), attention theory (Ocasio, 1997), theory of organizational ecology (Heine & Rindfleisch, 2013), and status theory (McDonnell & King, 2018)). Although there is some evidence in the literature for each of the aforementioned effects, the definition, description, and application given here are new. The identified forces and their corresponding effects show what can be called *a paradox of ethics*: the more ethical an organization becomes, the higher, in some respects, is the likelihood of unethical behavior. Unethical behavior is thus not the result of the organization being a bad barrel but of the organization becoming and being a good one.

This article differs from other studies on why good or excellent organizations do bad things (e.g., Greenwood, 2004; Mishina et al., 2010; Taylor, 2006). Such studies define “good” or “excellent” in economic, financial, or strategic terms. This article, however, will define good in ethical terms and considers this as part of the explanation for bad things, i.e., unethical behavior. This article is consistent with studies on other topics that use a similar approach to study how improving an object may trigger factors that cause the object’s own deterioration. For example, Methot et al. (2017) show how employees becoming good citizens may give rise to circumstances for becoming a morally worse citizen, thus resulting in lower organizational citizenship behavior.

The rest of the article is organized as follows. After defining what this article means by an organization becoming more ethical and looking at how the literature on organizational life cycles provides us a lens to understand the emergence and intensification of threatening forces and effects, the next section presents an overview of the four forces that emerge and intensify when an organization becomes more ethical, their corresponding effects and the theories that explain these effects. In the following sections, each of the four threatening forces is discussed and illustrated using two corresponding effects. The description of each effect follows this sequence: defining the effect, illustrating the effect with existing studies, explaining the effect with a theory, explaining how the effect is suggested to increase the likelihood of unethical behavior, and concluding with a proposition. The article ends with a summary and discussion of seven research and two practical implications.

## A Model of Threatening Forces and Effects

To identify factors that are activated and strengthened when an organization becomes more ethical, we first must make clear what it means when an organization becomes more ethical. Following the approach of the organization viewed as a barrel (Treviño & Youngblood, 1990), the ethical content of an organization is the extent to which the organizational context prevents employees to behave unethically (Kahn, 1990). Different models and scales have been developed to assess the organizational context in this regard. For example, Victor and Cullen (1988) have developed a five-dimensional model and scale to assess the ethical climate of any organization, and Kaptein (1998, 2008) has develop an eight-dimensional model and scale to assess the ethical culture of any organization. Many studies show that the more ethical the climate and culture of organizations are, i.e., the barrel becomes better, the less likely their employees behave unethically (Kaptein, 2011; Treviño et al., 2014). Unethical behavior is then usually defined in terms of behavior that is morally unacceptable to the larger community (Jones, 1991).

To better understand the dynamics of what happens when an organization becomes more ethical, we can use Reidenbach and Robin’s (1991) model of corporate moral development. Reidenbach and Robin, who are inspired by the work of Lawrence Kohlberg, distinguish five stages (or levels) in the moral development of organizations: the amoral, legalistic, responsive, emerging ethical, and the ethical organization. The higher the stage, the more the ethical culture and climate of the organization stimulates its employees to make ethical decisions. To operationalize their model, Reidenbach and Robin offer, without testing, several propositions. Examples of these propositions are: not all organizations pass through all stages of moral development; an organization can begin its life in any stage of moral development; moral development does not have to be a continuous process; an organization comprised of multiple departments can occupy different stages of moral development at the same time; there is no time dimension associated with the moral development of an organization; and two organizations can be in the same stage but one may be more advanced. Another proposition they offer is especially relevant to our article. Reidenbach and Robin propose that organizations at one stage of moral development can regress to lower stages. For them, regression “typically occurs because the concern for economic values is not adequately counterbalanced by the concern for moral values” (1991, p. 274). They add that new management or mergers and acquisitions can also provide an impetus for regression. Although Reidenbach and Robin discuss in their article the dynamics of the moral development of organizations, they do not examine the causes of these dynamics. Neither do they refer to the rich and useful literature on organizational life cycles, which was already available at the time of their article’s publication.

The literature on organizational life cycles is helpful for understanding the dynamics of the ethical development of organizations and for the purpose of our article: how this ethical development can activate threatening factors toward itself. The concept of organizational life cycle started with the biological analogy that organizations like natural organisms go through the process of inception to growth, maturity, decline, and death (Boulding, 1950; Marshall, 1890). To date, at least 45 different organizational life cycle models have been developed (Al-Taie & Cater-Steel, 2020). These models have in common that they distinguish different phases of organizational development, each phase having different opportunities, issues, needs, criteria and priorities (Gupta & Chin, 1994; Smith et al., 1985; Whetten, 1987). Research also suggests that threatening factors in the external and internal environment of an organization vary with the stages in life cycle (Jawahar & McLaughlin, 2001). These threatening factors can be activated and strengthened by the development of an organization. For example, Jawahar and McLaughlin (2001) show that specific stakeholders are likely to become more important as an organization evolves from one stage to the next and that the organization is at risk when it does not adapt its strategy to these stakeholders. In most models, the life cycle does not end with the death of organizations because organizations, after a decline, can also redevelop, revive, and renew itself.

**Fig. 1:**
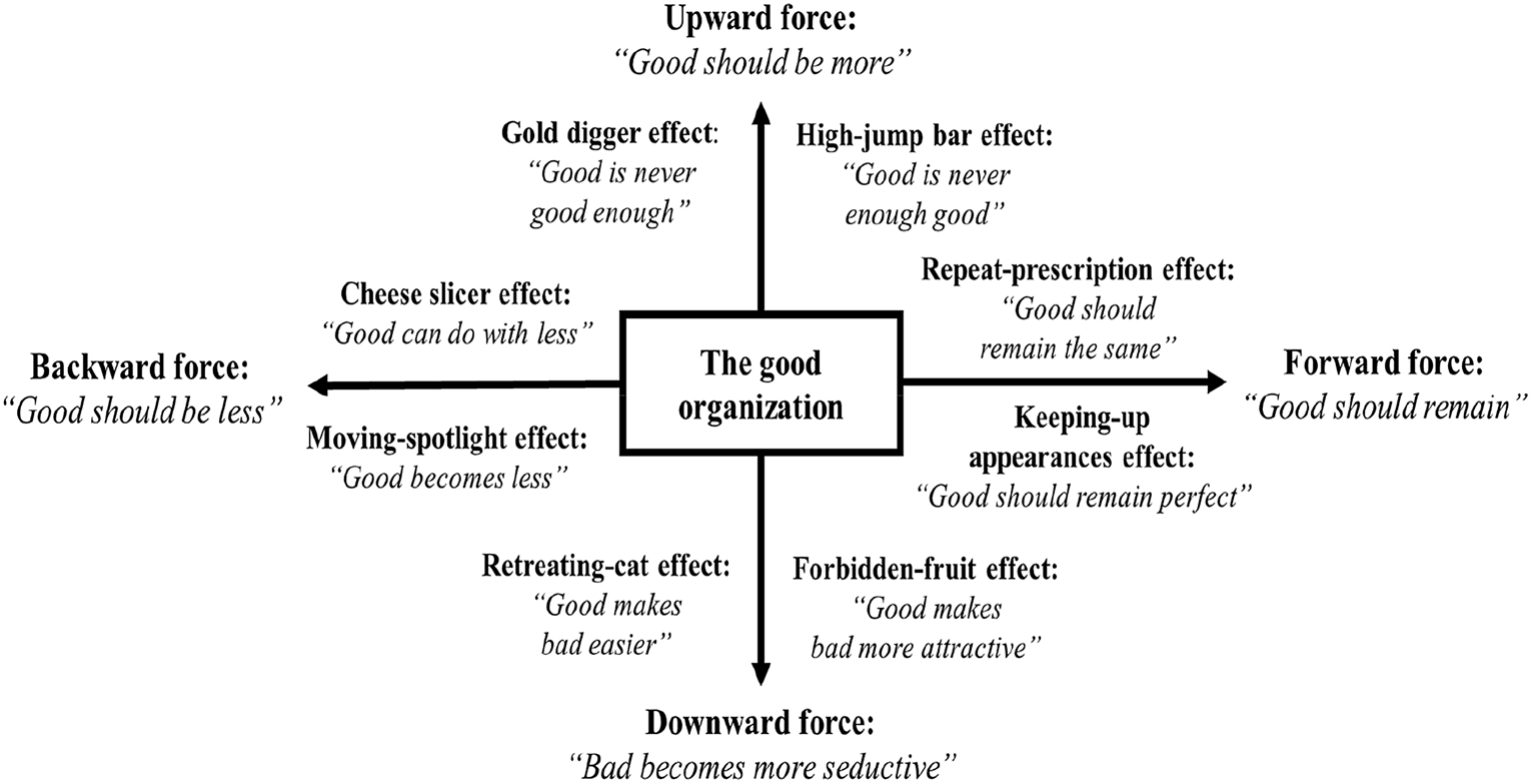
Model of threatening forces and effects on the ethical organization

This article looks for threatening factors inside and outside the organization, which are activated when the organization becomes more ethical. Combining the concepts of Reidenbach and Robin (1991) and of organizational life cycle (i.e., respectively, the ethics of an organization can develop dynamically, and threatening factors can be activated when an organization develops), we create a lens for examining, when the organization becomes more ethical, whether and which factors may be activated that increase the likelihood of unethical behavior. To explore whether the ethical development of organizations may activate threatening, countervailing factors, we start with which forces in an around organizations can arise when organizations become more ethical. These forces are the expectations, beliefs, and tendencies in and around organizations (cf. Lewin, 1951; Porter, 1980; Stead et al., 1990). The forces are first described here briefly and in general terms, and in the rest of the article, each is described in detail by means of two of their corresponding effects. These effects are mechanisms, patterns, or laws that may be discerned in practice. Figure 1 shows the forces and the corresponding effects that illustrate each of them. The forces are not bad or unethical as such, but their corresponding effects may lead to unethical behavior.

Four forces can be identified that may threaten the ethics of an organization when an organization becomes more ethical. The first force is about the expectation, in and around organizations, that the organization should become even more ethical. The belief that something good can and should be better triggers this force. This force can be called an upward force because the expectation is that the ethics of an organization should go “upward.” A second force is in the opposite direction in the sense that it tries to pull down the ethics of an organization. This so-called downward force is about unethical behavior becoming more seductive when an organization become more ethical. This force is triggered by the belief that the better something is, the more tempting badness becomes. The third force is about the expectation that the organization should reduce its investments in ethics. This force is triggered by the belief that when something is good, it can and should stay good with less resources. This force can be called a backward force because it pressures the organization to go back to the time when the organization was less ethical and paid less attention to ethics. The fourth force is in the opposite time direction in the sense that is it about staying the same in the future. This so-called forward force is about the expectation that the organization should continue its ethics and not change it. This force is triggered by the belief that when something goes well, it should remain well.

These threatening forces create a paradox of ethics. Each of these forces pulls, with their corresponding effects, on the ethically good organization such that when the ethical level of an organization improves, these forces become stronger. Because they increase the likelihood of unethical behavior in an organization, when these forces and their corresponding effects become stronger, the likelihood of unethical behavior also increases in this respect. This is a paradox of ethics: certain threatening forces arise when an organization becomes more ethical, thereby increasing the likelihood of unethical behavior. It is a sad paradox because it is about goodness breeding badness. This paradox fits in the literature on organizational paradoxes (Berti & Simpson, 2021), where a paradox is defined as “contradictory yet interrelated elements that exist simultaneously and persist over time” (Smith & Lewis, 2011, p. 382). More specific, the proposed paradox of ethics belongs to the type of paradoxes that Putnam (1986) identifies as self-referential loops or double binds about the ironic or contradictory outcomes arising from a specific action.

In the next sections, each of the four forces is described by means of two effects that arise when an organization becomes more ethical. Each effect is defined and illustrated by existing studies, explained by a different theory, and suggested to increase the likelihood of unethical behavior in at least one way. Each effect thus leads to a proposition. Table 1 provides an overview of the definitions of the effects and the theories used. The effects are described here as being independent from each other. As proposed in the discussion section, studying their mutual relationships is a topic for future research.

**Table 1:** Overview of definitions of threatening forces and effects and theories used

Threatening force	Threatening effect	Definition	Theory
Upward force: Ethics should be more	Gold digger effect	The more ethical an organization becomes, the more its imperfections are scrutinized until they are found	Social identity theory
	High-jump bar effect	The more ethical an organization becomes, the higher the ethical standards are set until they cannot be met	Moral progress theory
Downward force: Unethical behavior becomes more seductive	Retreating-cat effect	The more ethical an organization becomes, the more the oversight on the ethics of the organization decreases until this situation is abused visibly	Theory of organizational trust
	Forbidden-fruit effect	The more ethical an organization becomes, the more attractive unethical behavior becomes until it could not be resisted	Forbidden-fruit theory
Backward force: Ethics should be less	Cheese slicer effect	The more ethical an organization becomes, the lesser the investment in ethics until the investment is no longer enough	Resource dependence theory
	Moving-spotlight effect	The more ethical an organization becomes, the more the focus is on what is *not* good, until what is good is no longer good as a result	Attention-based view of the firm
Forward force: Ethics should remain	Repeat-prescription effect	The more ethical an organization becomes, the longer its way of managing ethics continues until it becomes outdated	Theory of organizational ecology
	Keeping-up appearances effect	The more ethical an organization becomes, the more defects are disapproved of and hidden until they cannot be hidden anymore	Status theory

## The Upward Force

The upward force on the ethical organization is the expectation that the more ethical an organization becomes, the more it should become more ethical. The belief that something good can and should be better triggers this force. “Good should be more” is the mantra that expresses this force. Something can always be more ethical; the ethics of an organization can always be better. Even when the organization is ethical, the expectation is that the organization should further improve its ethics; the present ethics of the organization is never sufficient. This is the demand that the ethics of an organization should go “upward.” Two effects illustrate this force: the gold digger effect and the high-jump bar effect.

### The Gold Digger Effect

The gold digger effect means that the more ethical an organization becomes, the more critically and intensively imperfections are sought until these are found. This effect is based on the expectation that imperfections—for example, an ethical policy that is not fully embedded or an ethical standard that is not fully complied with—are perceived by the “diggers” as “gold”; such imperfections have a great value and take time and energy to be discovered. The “diggers” refer to evaluators within the organization (such as managers, compliance officers, and auditors) or outside it (like inspectors, regulators, raters, accountants, journalists, and activists) who inspect, check, assess, monitor, and investigate the ethics of the organization (cf. Aguilera et al., 2015). They are prompted by the idea that no matter how good an organization is, “Good is never good enough.” An organization is never perfect: there will always be a gap, defect, or deviation to be found, and this is just a matter of thorough and meticulous searching. This is like gold panning: the process of finding gold by carefully and repeatedly sifting alluvial deposits. However, the more ethical an organization becomes, the less easy it is to find imperfections, and so the search takes longer.

We can find indications of the gold digger effect in research. Kassin et al. (2003) found that as long as suspects do not confess, forensic interviewers try harder to obtain evidence and increase the pressure so the suspects would confess in the end even when they are innocent. McMillan and White (1993) found that auditors who believe that they have to uncover material errors in their clients’ financial statements continue their search for such errors, even when their initial search indicated otherwise, and they start to look for irrelevant errors. The gold digger effect is also suggested by Strickland’s (1958) reference to managers who, because they distrust their employees, check the behavior of their employees until they find evidence that the employees indeed cannot be trusted. Other examples of the gold digger effect are doctors examining healthy patients until they find an issue and label them sick (cf. Kerr, 1975), car mechanics looking for a deficiency in a well-functioning car until they find and can repair it (Schneider, 2012), or police officers who surveil citizens until they can issue a fine (Hoogenboezem & Hoogenboezem, 2005).

Social identity theory is useful in explaining the gold digger effect. This theory holds that how people view and define themselves and how they interpret the world influence their behavior (Albert & Whetten, 1985; Ashforth & Mael, 1989; Ashforth & Schinoff, 2016). Both professional and organizational identities are relevant for explaining the gold digger effect. What is considered in the profession and in the organization of ethics evaluators to be a good and successful evaluator influences what is perceived to be a good and successful evaluation. For example, auditors who view themselves as error finders would value professional skepticism and conservative behavior (Smith & Kida, 1991) and define their success in terms of the errors they detect. This error-finder identity is also fueled by the conception that evaluators are guardians who catch abuses of trust (Braithwaite, 1998). The success of an evaluator may then be expressed in the number of detected violations, incidents, and deficiencies. Especially when the organization is perceived by others to be good, evaluators can show their value and skills by uncovering the organization’s ethical deficiencies. So, when the identity of evaluators is related to the imperfections they discover, this may lead to the situation that the more ethical an organization becomes, the harder it is to find imperfections, the more the evaluator would want to find imperfections, and the more esteemed the evaluator is when they do find imperfections.

The gold digger effect may increase the likelihood of unethical behavior in organizations by creating negative emotions among the people within the organization. Evaluators believing that an organization is never good enough—even when the organization is actually ethical and without any imperfection—can lead to negative emotions among managers and employees: that their efforts are not seen, appreciated, and counted. For example, Belschak and Den Hartog (2009) show that negative feedback leads to more counterproductive behavior and less organizational citizenship behavior because negative feedback induces negative emotions (e.g., anger and frustration) among employees, who then redirect their attention toward the object of the feedback (in our case, the imperfections) and away from what the focus should be (in our case, the ethical development of the organization). The gold digger effect may lead to the fear within an organization that no matter how ethical the organization performs, this is never sufficient, and someone can always be blamed. This fear can make managers and employees reluctant to take new initiatives and accept responsibilities. The negative emotions of feeling misidentified (Meister et al., 2014) can also be directed at the giver of the feedback (in our case, the diggers). When these diggers are portrayed as opportunistic and only want to find something negative, this can foster a spiral of distrust (Acemoglu & Wolitzky, 2012) that may lead to less support within the organization for the diggers, for the ethical interests and standards they represent, and for their findings and recommendations (cf. Quintelier et al., 2018). Consequently, less support for ethics may lead to an increase in unethical behavior within the organization, given that support for ethics is a main driver of ethical behavior and a key virtue of the ethical organization (Kaptein, 2011, 2017).

#### P1

The more ethical an organization becomes, the more its imperfections are sought, thus making unethical behavior by employees more likely.

### The High-Jump Bar Effect

The high-jump bar effect means that the more ethical an organization becomes, the higher the standards are set until they could no longer be met. This second effect of the upward force is based on the expectation that every time an organization “jumps over the bar” (i.e., meets an ethical standard, norm, or rule), the bar is raised, i.e., the standard, norm, or rule is raised by the organization itself, by its stakeholders, or by society. For example, when an organization has successfully implemented the norms for its employees regarding insider trading, it is then expected that it also defines and implements them for the family members of its employees. The idea behind the high-jump bar effect is that if an organization functions well, it can always do better, go a step further, do more than expected. “Good is never enough good.” If the organization can bear the current responsibilities, then extra or new responsibilities can be added. This line of thinking means that standards are raised until they are too high that the organization could not meet them anymore and thus fails.

Research suggests elements of this effect. The high-jump bar effect resembles the Peter Principle, which says people are promoted to their level of incompetence (Lazear, 2004; Peter & Hull, 1969), and the idea that when people have attained a goal, they typically set a higher one (Locke & Latham, 2019). The same holds for organizations: organizations raise their performance targets when these are met; goals accumulate, are raised, and stretched (Sitkin et al., 2017). The result is a struggle to meet the bar (Schaubroeck et al., 2021). The high-jump bar effect also reflects the ever-expanding circle of moral concern (Singer, 1981), the irreversible moral development of society (Moody-Adams, 1999), the growing number of laws and regulations for organizations (Martinez-Moyano et al., 2014; Short & Toffel, 2010), the broadening of the moral responsibilities of organizations (Eberlein, 2019; Mayer, 2021), and the new norms organizations frequently introduce in their code of ethics (Singh et al., 2011).

The theory of moral progress is useful in explaining the high-jump bar effect. Kaptein (2021) explains that the moral progress in organizations is gradual and stepwise because an organization’s moral responsibility to adopt a new or higher norm depends on the corporate resources. Adopting and implementing a norm requires resources, and organizations cannot be responsible for matters for which they do not have the resources. However, Hahn et al. (2016) argue that organizations achieve higher levels of corporate social performance through the ambidextrous ability to simultaneously pursue instrumentally and morally driven social initiatives. Kaptein (2021) appeals to this argument to contend that once a norm is implemented and becomes integrated into the core routines of an organization and subjected to a commercial logic, the norm releases and creates resources that can be used to introduce a new or even more demanding or stricter norm. For example, Tanyi and Litt (2017) found that the implementation of higher audit quality standards within auditing firms led to higher auditing fees for clients. Short and Toffel (2010) found that the more standards an organization implements successfully, the easier it becomes for the organization to implement a new standard. This is because by successfully implementing a standard, an organization develops what they call a good compliance routine of accumulated encoded organizational capabilities and knowledge. The high-jump bar effect is set in motion by the belief—within and outside organizations—that successfully implementing an ethical standard makes an organization better able to adopt and to implement a higher or new standard.

The high-jump bar effect may increase the likelihood of unethical behavior in an organization when the standards are set too high, and the organization can no longer meet them. When the only way to know an organization’s maximal absorptive capacity is by implementing ethical standards until the organization can no longer implement them well, this means that standards are set too high, beyond the organization’s capacity to be able to implement them successfully, thus potentially leading to a rise in unethical behavior in the organization. Furthermore, the frequent introduction of new standards can lead to standard erosion. While Short and Toffel (2010) find that the successful implementation of standards increases the commitment of organizations to self-regulate, the opposite can also be the case. Goal erosion can occur when goals are set too high, and the goal commitment decreases (Koo & Fishbach, 2010). Standard erosion is akin to this and means that people within organizations become less committed to ethics when they believe that the introduction of new standards is never-ending and that these standards are never sufficient. The decrease of commitment to ethics may increase the likelihood of unethical behavior given that the commitment of employees is essential for preventing unethical behavior (Kaptein, 2011). This effect is especially threatening when managers and employees anticipate this effect by suboptimally implementing a standard to avert the introduction of a new standard. The suboptimal implementation of the standard then risks causing unethical behavior in the organization.

#### P2

The more ethical an organization becomes, the higher are the ethical standards set, thus making unethical behavior by employees more likely.

## The Downward Force

The downward force on the ethical organization refers to the expectation that the more ethical an organization becomes, the more seductive unethical behavior becomes. This force is triggered by the belief that the better something is, the more tempting badness becomes for it. “Bad becomes more seductive” is the mantra that expresses this force. Unethical behavior becomes more attractive when an organization becomes more ethical. This force tries to pull down the ethics of an organization. Thus, whereas the upward force is about the ethics of an organization becoming better or higher, the downward force is about the ethics of an organization becoming worse or lower. Two effects that illustrate this force are the retreating-cat effect and the forbidden-fruit effect.

### The Retreating-Cat Effect

The retreating-cat effect means that the more ethical an organization becomes, the more the oversight on the ethics of the organization decreases until this situation is visibly abused by people within the organization. This effect, which is the opposite of the gold digger effect, is based on the expectation that those who surveil, supervise, and inspect an organization (the proverbial cats or watchdogs) will reduce the frequency and thoroughness of their monitoring of the organization, i.e., retreat from the organization, when the organization becomes more ethical. However, the more the cat retreats, the more the “mice” (the people within the organization) can exploit this situation and be tempted to behave unethically. For example, when the ethical level of an organization is unsatisfactory, compliance officers, controllers, and regulators increase their oversight; and when the ethical level meets their expectations, they relax their oversight until they learn about the abuse, and then they intensify their oversight. This is analogous to placing speed control cameras when and where cars drive too fast, removing them when cars keep to the speed limit, and then putting the cameras back when there are indications that cars are again driving too fast. The paradox of the retreating-cat effect is that goodness promotes badness because “good makes bad easier.” This retreating-cat effect differs from Braithwaite’s (2002) and Ozcan and Gurses’ (2018) concept of cat-and-mouse play. In their concept, the cats are the regulators, while in the retreating-cat effect, the cats can be all stakeholders that supervise the organization, like investors. Furthermore, their concept is about organizations that are trying to escape from the cat, while the retreating-cat effect starts with the idea of the cat that is leaving.

We can find elements of the retreating-cat effect in studies that show that the discovery of unethical practices in organizations leads to more monitoring, inspections, checks, surveillance, and supervision by outsiders of those organizations. For example, Zahra et al. (2005) describe how at the start of this century fraud scandals by top management led to more and closer oversight among all kinds of stakeholders, such as shareholders, debtholders, regulators, and auditors. Buyers who are betrayed by sellers can become more vigilant (Leonidou et al., 2017). Research also shows that oversight decreases when the situation is normalized. When an organization is accused of legal violations and it agrees to settle, regulators install a compliance monitor who independently oversees the design and implementation of a compliance program for the organization; the compliance monitor then retreats afterward once the organization has satisfactorily improved its compliance level (Ford & Hess, 2008). Another example is the concept of responsive regulation: a regulator bases its strategy on the self-regulation of the organization by taking distance when an organization is virtuous and committed to comply with regulations and becoming stricter, more stringent, and closely monitoring the organization when the latter is less virtuous and committed. In this case, the enforcement strategy of regulators is to follow the compliance level of the regulatees (Braithwaite, 2007, 2020).

The theory of organizational trust is useful in explaining the retreating-cat effect. Mayer et al. (1995) have developed a dynamic model of organizational trust in which three perceived characteristics of the trustee, i.e., ability, benevolence, and integrity, lead to the trustor trusting the trustee. In their model, which is supported by empirical evidence (Colquitt & Rodell, 2011), trust is seen as a substitute for monitoring. When trust is low (trustee is not perceived to be trustworthy), trustors will be more inclined to monitor the trustee so they can be confident that they are not being misused or exploited. However, when trust is high, trustors will be less inclined to monitor the trustee because the likelihood that they will be misused or exploited is less than when they do not completely trust the trustee. Furthermore, when trust is high, monitoring is not efficient because it requires resources (Jones & George, 1998), it is not viewed to be ethical because it shows a lack of respect for the efforts of the trustor (Braithwaite, 2020), and it is not effective because the trustee may interpret monitoring as a signal of mistrust that may breed distrust toward the trustor, retaliation, and unethical behavior (Ferrin et al., 2007; Mayer et al., 1995). This means that, as Ferrin et al. (2007) state, as trust increases, monitoring is obviated. The further implication of this is that the more ethical an organization becomes, in terms of ability, benevolence, and integrity, the more trustworthy and trusted the organization is, the less inclined others will be to monitor the organization; however, these others will be more inclined to intensify their monitoring when they see that the organization has abused their trust and its trustworthiness is lower than they had expected.

The retreating-cat effect may increase the likelihood of unethical behavior in organizations when there is increasing lack of oversight and unethical behavior is too tempting. This is because the likelihood that the unethical behavior will be detected declines, and so unethical behavior becomes easier and more profitable. Clarke (2012) finds that even law-abiding people can be seduced and overwhelmed by opportunities for criminal behavior. Opportunity makes the thief (Felson & Clarke, 1998; Gelter & Helleringer, 2018). Lesser monitoring decreases the expected costs of noncompliance (Andarge & Lichtenberg, 2020) and confronts people in the organization, even if it is ethical, with stronger temptations to commit unethical behavior (cf. Olekalns et al., 2020). Whereas transparency is an inhibitor of unethical behavior (Haack et al., 2021; Kaptein, 2011; Lehman & Ramanujam, 2009), a lack of transparency makes unethical behavior more tempting and increases the likelihood that people in the organization would not be able to resist these stronger temptations. The paradox is that the organization is monitored less by others due to the belief that the organization is trustworthy, thereby increasing the likelihood that members of the organization will misuse this trust by behaving unethically.

#### P3

The more ethical an organization becomes, the lesser its oversight, thus making unethical behavior by employees more likely.

### The Forbidden-Fruit Effect

The forbidden-fruit effect means that the more ethical an organization becomes, the more attractive unethical behavior becomes until it could not be resisted. This second effect of the downward force is captured by the expression, “Good makes bad more attractive.” The forbidden-fruit effect is present in the literature (e.g., Binder et al., 2020; Fitzgibbon et al., 2020). The name of the effect refers to the biblical story of the forbidden-fruit in paradise that became too tempting and the consumption of which had momentous consequences. Applying the forbidden-fruit effect on the ethical development of organizations means that when organizations become more ethical, doing something that is not allowed becomes more satisfying, reasonable, and lucrative. The more and the higher the organizational standards, rules, and procedures are and the better they are embedded in the organization, the more attractive it is for the members of the organization not to adhere to them.

Several studies have demonstrated the forbidden-fruit effect. Research outside the setting of organizations shows that prohibiting smoking, marijuana, cybersex, and graffiti makes them more attractive and more likely for people to engage in them (Gosselt et al., 2012). Research also shows that when dieters deny themselves a particular food, their desire for the forbidden food and the frequency of their thoughts about it increase (Mann & Ward, 2001). It has also been found that the more weight people lose with their diet program and the closer they get to their desired weight, the stronger the temptation to break their diets and the more they succumb (Armitage et al., 2014; Soetens et al., 2008). Research also shows that people who act ethically are more likely to consequently lie, commit fraud, and cheat (Blanken et al., 2015). In the organizational setting, research shows that the introduction of standards makes the violation of these standards more tempting and likely (Bachram, 2004; Martinez-Moyano et al., 2014). For example, Bennett et al. (2013) show how stricter vehicle emission tests made manipulating the results more attractive and profitable for test facilities so they could please customers whose vehicle would fail the test. Clark and Newell (2013) show how rating agencies developed high standards for giving credible information to investors but once these standards were established, inflated ratings started to increase in frequency because they became more profitable since their advice was trusted.

The forbidden-fruit theory is useful to explain the forbidden-fruit effect. Forbidden-fruit theory (Bushman & Stack, 1996) encompasses commodity theory. The latter holds that the more a commodity is perceived to be unavailable or not easily obtainable, the more it is valued compared to a commodity that is freely and easily obtainable. Fitzgibbon et al. (2020) argue that forbidden options make people infer hidden value in them because they assume that there must be a good reason why the item is forbidden. The forbidden-fruit theory also encompasses reactance theory that holds that the more something is forbidden, the more this is perceived to threaten people’s freedom, thus the more satisfying it is to do what is forbidden because this restores people’s freedom, autonomy, and self-determination (Brehm, 1966). The forbidden-fruit theory applied to the ethics of organizations means that the more ethical an organization becomes, the more types of behavior are not allowed or practiced because these are defined as unethical; and so the more tempting it is to do what is not allowed and practiced.

The forbidden-fruit effect may increase the likelihood of unethical behavior in organizations when people within an organization cannot resist the increasing attractiveness of such behavior. When the temptation to behave unethically becomes stronger, it becomes more likely that people will succumb to it, i.e., behave unethically (cf. Baumeister et al., 2001). By doing what is forbidden, people can derive satisfaction from doing it, like getting a kick from carrying out prohibited transactions, embezzling funds, or selling bad products. Moreover, the longer the exposure to the temptation, the more likely people will succumb because their self-control is depleted (Gino et al., 2011). By tasting once what is forbidden, people run the risk that they would want to taste some more, get on the slippery slope (Rose et al., 2021), and thus make unethical behavior normal again. Tragically then, the resulting unethical behaviors are triggered by the organization becoming more ethical.

#### P4

The more ethical an organization becomes, the more attractive unethical behavior becomes, thus making unethical behavior by employees more likely.

## The Backward Force

The backward force refers to the expectation that the more ethical an organization becomes, the less the organization invests in ethics. This force is triggered by the belief that when something is good, it can and should stay good with less resources, time, energy, and effort. “Good should be less” is the mantra that expresses this force. The organization should stay ethical but not at any price and only with the necessary investments. So, the higher the ethical level of an organization, the stronger is the pressure to economize on ethics, to save on ethics management, and to focus only on what is not yet ethical. This is a backward force because by exerting pressure on the organization to cut back on their investments in ethics, it pressures the organization to go back to the time when the organization was less ethical and invested less in ethics management. Two effects that illustrate this force are the cheese slicer effect and the moving-spotlight effect.

### The Cheese Slicer Effect

The cheese slicer effect means that the more ethical an organization becomes, the more the organization reduces its investments in ethics until the reduction is too much. Allen and Imrie (2016) use the metaphor of a cheese slicer to explain how governments relentlessly, slice by slice, cut back budgets of the research sector. Applied to the ethics of an organization, this metaphor refers to an organization repeatedly slicing off its investments in ethics. For example, an organization periodically reduces its compliance budget, time spent on ethics training, and the scope of its ethics risk assessment. “Good can do with less” captures this effect. The idea behind the cheese slicer effect is that for as long as it goes well, the organization can invest less time, money, and effort on ethics because the organization should not be too ethical anyway, or be ethical at any price, or to invest too much in ethics.

There are references to the cheese slicer effect in research. People tend to postpone a routine dental or car maintenance until there’s toothache or their car breaks down (Consumer Reports National Research Center, 2011; Kranz et al., 2021). Research also shows that organizations tend to postpone maintenance of new buildings until the facilities are unhealthy and unsafe (Hamid et al., 2007), and that when downsizing their organization, managers tend to reduce the number of employees until there is insufficient people left and problems expand (cf. Williams et al., 2011). Postponing preventive maintenance activities of systems can also be a deliberate strategy of organizations: as they wait for incidents, which initially leads to higher costs, additional information enables more effectively planned maintenance activities during the remainder of the system’s lifespan (De Jonge et al., 2015). Martinez-Moyano et al. (2014) show in their research that once transaction compliance was achieved within financial firms, revenue producers increased pressure on compliance professionals to divest from compliance because compliance undermined the competitive advantage and profitability of their firm. In the specific case of Siemens, Eberl et al. (2015) show that Siemens implemented all kinds of rules after their prominent corruption scandal and then gradually relaxed these rules to give their employees more room to conduct business. The CEO at that time explained this as “the pendulum has swung too far; we have to find the center again.” (p. 1217).

The resource dependence theory is useful for explaining the cheese slicer effect. Durand et al. (2019) use the resource dependence theory to model how organizations respond to normative pressures. The level of compliance with ethical norms depends on the ability of organizations to comply with the norms. This ability consists of the decisions makers’ (i.e., organization’s) perceived costs and benefits of complying with the norm. The higher the net benefit, the more likely the organization will comply. However, as resources are scarce, organizations have to allocate their resources as efficiently as possible to comply with the norms and at the same time have sufficient resources to invest in other activities needed to meet the objectives of the organization. Therefore, investing too much resources on ethics, i.e., more than is needed to comply with ethical norms, is a waste of resources, which would lead to higher costs but no benefits. So apart from the questions whether ethics pays (Lynn, 2020; Schwab, 1996) and whether it may lead to lower costs (Jones, 1995), organizations tend to reduce their investments in ethics to the level where they remain ethical without wasting resources. By divesting from ethics but remaining ethical, the organization is synthesizing economics and ethics (Jones, 1995) and integrating profit and principles (Graafland, 2002). However, finding the optimal level of investment is difficult because the costs precede the benefits, there are diminishing marginal returns to ethics, and it can be difficult to establish the costs for becoming and remaining ethical. In addition, the cost–benefit ratio of the investment in ethics is rather intangible because the costs are difficult to calculate ex ante and the benefits are uncertain and difficult to attribute ex post (cf. Durand et al., 2019; Flammer, 2013; Wickert et al., 2016). As a result of all these difficulties, an organization reduces its investments in ethics slice by slice, step by step, to make sure that the reduction will not be too big at any moment.

The cheese slicer effect may increase the likelihood of unethical behavior in an organization when the organization divests too much from ethics. The risk of the cheese slicer effect is that an organization slowly reduces its investment in ethics until the frequency of unethical behavior in the organization starts to increase. By the time an unethical behavior, which could have been prevented by additional investment in ethics, occurs, the organization knows it has divested too much (cf. De Jonge et al., 2015). However, when those who decide on the investments in ethics remain ignorant of this unethical behavior, then an organization runs the risk of further divesting from ethics because it wrongly assumes that the organization’s ethics is still healthy. However, when the deterioration of ethics becomes known to such decision makers, the frequency of unethical behavior can still increase further when the implementation of new activities to restore the ethics of the organization is delayed. This can happen because defining what is needed, allocating resources, and embedding new activities in the organization take time (Hoekstra & Kaptein, 2021).

#### P5

The more ethical an organization becomes, the more it reduces its investments in ethics, thus making unethical behavior by employees more likely.

### The Moving-Spotlight Effect

The moving-spotlight effect means that the more ethical an organization becomes, the more the focus is on what is not good, until what is good becomes not good as a result. This second effect of the backward force is based on the belief that the attention both inside and outside an organization is more on what needs to be improved than on what has been improved, on what is insufficient than on what is sufficient, and on what the current issue is than on what the issue was in an organization. When an organization becomes more ethical, what gets attention, i.e., what is in the spotlight, changes. For example, when an outdated code of ethics is updated, the content of the code is no longer on the agenda of management; this is replaced by the implementation of the code. When the frequency of fraud is reduced to acceptable levels, the attention of management and stakeholders moves to reducing another type of frequently occurring unethical behavior. The moving-spotlight means that what comes under the spotlight gets more attention and what goes out of the spotlight gets less attention than they did before. In this sense, “Good becomes less”: less salient, less interesting, and less topical. The moving-spotlight effect is different from the spotlight effect identified by Gilovich and Savitsky (1999). The latter is about the tendency of people to believe that they are being noticed more than they really are.

There are references to the moving-spotlight effect in research. Jones et al. (2017) found that schools tend to focus on the topics their inspectors focus on, with all kinds of negative effects. “What gets attention grows” is a popular management expression and it means what does not get attention stops growing or even shrinks (Scandura & Gower, 2019). Briscoe et al. (2015) found that in the wake of heightened issues, organizational decision makers move their focus on events related to the issues to explore the possible merits and methods of responding to those events. Baldwin and Black (2016) show how regulators move their focus by using risk-based and problem-centered techniques to identify, select, and prioritize the issues they focus on when they supervise organizations. In a similar vein, Aguilera et al. (2015) show how the media is selective in putting issues on the public agenda, while McDonnell et al. (2015) show this same selectiveness on the part of NGOs. Moreover, at a broader, societal level, which issues come to be in the spotlight change. For example, before the COVID-19 pandemic, work-related issues like #Metoo and Black Lives Matter were high on the society’s agenda. After the pandemic, more attention were given to issues like hybrid working, inequality and animal trafficking (Bapuji et al., 2020; Carroll, 2021).

The attention-based view of the firm is useful for explaining the moving-spotlight effect. The attention-based view of the firm (Ocasio, 1997, 2011) holds that organizations focus and distribute their attention. Because human and organizational attentional capacity is limited—i.e., it is not possible to pay attention to everything that is relevant—people and organizations should be selective in their attention. This attention is, according to Durand et al. (2019), important in explaining the level of compliance by organizations. As managerial attention is limited, some issues may be evaluated by managers as being either more or less salient than others. The more salient an issue is evaluated to be, the more likely an organization will address it by implementing the related standards. What Durant and colleagues fail to highlight is that the question of salience also applies to stakeholders: in trying to bring the attention of an organization to the issues, the stakeholders, given their limited attentional capacity, cannot pay attention to all the concerns or questions (cf. Dutton & Ashford, 1993). Limited attentional capacity helps explain the life cycle of issues. The attention for issues grows and goes (Mahon & Waddock, 1992) because not every issue can get all the attention. That attention is selective implies that the more an issue is in the spotlight and, as a result, gets addressed, the more opportune it becomes to move the spotlight on another issue. By addressing a new concern, the organization can further improve its ethics and show its proactive commitment to it (cf. Kaptein, 2019; Solinger et al., 2020).

The moving-spotlight effect may increase the likelihood of unethical behavior in organizations when an ethical issue that is not in the spotlight gets less attention and fades into the background until it becomes an issue again. Addressing an ethical issue successfully requires less attention from management (Short & Toffel, 2010). However, there is the risk that because the maintenance and safeguarding of the ethical issue gets too little attention and insufficient time and energy from management, unethical behavior is likely to increase. Paying sufficient attention to previous issues is important to maintain strong awareness and commitment for each of those issues within the organization (Hoekstra & Kaptein, 2021). The paradox, however, is that due to the ethical development of an organization, the issues that have been well addressed get less attention now and consequently, the likelihood of unethical behavior regarding those issues increases. Indeed, when this happens, the issue may again become current and so move up on the management’s agenda. This explains why the life cycle of ethical issues is not one-off but cyclical, and why issues such as fraud and corruption may be high on the agenda of organizations every few years.

#### P6

The more ethical an organization becomes, the more it focuses on new ethical issues, thus making unethical behavior by employees regarding earlier issues more likely.

## The Forward Force

The forward force is the expectation that the more ethical an organization becomes, the more it should continue on its ethical path. This force is triggered by the belief that when something goes well, it should remain well. “Good should remain” is the mantra that expresses this force. What is good should not change. This is a forward force in the sense that it concerns ethics in the future. Thus, while the backward force is about going back in time by investing les, the forward force is about staying the same in the future. The two effects that illustrate this force are the repeat-prescription effect and the keeping-up appearances effect.

### The Repeat-Prescription Effect

The repeat-prescription effect means that the more ethical an organization becomes, the longer it continues with the same way of managing ethics, until this becomes outdated. This repeat-prescription effect concerns the pressure and tendency inside and outside organizations to continue with what is going well until it no longer does. “Good should remain the same” is the mantra that expresses this effect. For as long as things are going well, there is no reason to change anything. This is like prescribing the same medicine for as long as the illness does not worsen, until the patient becomes resistant to it and the illness recurs. Applied to organizations, this effect means that organizations manage their ethics in the same way to mitigate unethical behavior, until this way becomes less effective and unethical behavior becomes more frequent and serious. For example, an organization employs every year the same e-learning in ethics for all its employees because the e-learning was initially well received, until the employees are no longer responsive when going through the same e-learning. Or an organization applies its ethics program to every new ethical issue in the same way because the program and process worked well for previous issues, until this style fails to effectively or adequately address a new issue. The repeat-prescription effect suggests that the more successfully an organization manages ethics, the more likely the organization would not change and would simply continue with this way of managing.

We can see elements of the repeat-prescription effect in research. Studies on the paradox of success explain why and when success breeds failure (Iso-Ahola & Dotson, 2014). A study in the airline and trucking industries by Audia et al. (2000) show that greater past successes of organizations subsequently led to performance decline. Miller (1992) calls this phenomenon the Icarus paradox: when organizations abruptly fail after a period of apparent success, brought about by the very elements that led to their initial success. There is Sull’s (1999) in-depth report about how the success of the rubber company Firestone led to its decline due to management holding on to their success formula for too long, despite indications that this formula had become outdated. The mantra, “Never change a winning team” (Dilger, 2002) is also indicative of this effect in the sense that a team is changed only after it has lost. McKinley et al. (2014) argue that organizations may become successful through their innovations, then slow down once they are successful and only accelerate innovation when their performance declines. Hogenbirk and Van Dun (2021) find that on average, the ethics programs and officers they have studied could be much more innovative. They recommend periodically changing and expanding ethics programs for these to remain effective, which requires ethics officers to be innovative.

The theory of organizational ecology is useful for explaining the repeat-prescription effect. Organizational ecology highlights the way in which organizational patterns change over time when organizations are under pressure from an ongoing selection process (Heine & Rindfleisch, 2013). One important finding of organizational ecology is that inert organizations have a higher probability of surviving than organizations that frequently try to adapt to their environment (Heine & Rindfleisch, 2013). Stable organizations have built certain core features and resources that provide reliability and accountability, which stakeholders value. However, from the perspective of organizational ecology, organizational inertia plays an ambiguous role; it is a double-edged sword (Audia et al., 2000). Organizational inertia also accounts for an organization’s inability to adapt to new challenges: the organization’s stability leads to a persistent resistance to change. Sull (1999, 2005) explains that good companies become bad and success can eventually breed failure due to active inertia, which is not about an organization’s inaction. Active inertia is the organization’s tendency to repeat the activities that contributed to its past success because these activities are now second nature. The concept of organizational inertia applies to the financial and competitive performance of organizations (Heine & Rindfleisch, 2013; Sull, 1999), but it can also be applied to the ethical development of organizations. When an organization becomes ethical, it means it has found a way, a routine or formula, to achieve this: by defining, embedding, and safeguarding ethics in a certain way. The more successful an organization’s management of ethics is, the less likely will the organization change it. Managing the ethics of an organization is a complex balancing act (Kaptein & Wempe, 2002), so changing the way of managing ethics, even if it is to address current or new issues, means risking that this balance will be disrupted; however, avoiding this risk weakens the readiness of an organization to change its way of managing ethics.

The repeat-prescription effect may lead to unethical behavior in organizations when the unchanging way of managing ethics becomes less effective in preventing, detecting, and responding to unethical behavior. By managing ethics in the same way, an organization risks its management of ethics becoming outdated due to, for instance, the organization’s environment changing. For organizational ecology theory, this is the major explanation for the decline of successful organizations: new competitors, new customers, and new markets (Heine & Rindfleisch, 2013; Miller, 1992; Sull, 1999). From an ethics management perspective, new stakeholders, new issues, and new ethical risks may require changes in the ethics program of an organization (Hogenbirk & Van Dun, 2021; Kaptein & Wempe, 2002). Despite increasing pressure to change a continuingly successful ethics program, organizations may not to do so, until their ethics deteriorates. For example, the organization’s e-learning program is only renewed when it becomes clear that it is seriously outdated. This echoes McKinley et al.’s (2014) observation that certain organizations only start to innovate when they are in decline. The paradox of the repeat-prescription effect is that what once worked well to improve the ethics of an organization is the very thing that leads to its deterioration, thus making unethical behavior in the organization more likely.

#### P7

The more ethical an organization becomes, the longer it continues the same way of managing ethics, thus making unethical behavior by employees more likely.

### The Keeping-Up Appearances Effect

The keeping-up appearances effect means that the more ethical an organization becomes, the more the defects in the organization are disapproved of and thus, hidden, until they cannot stay hidden anymore. This second effect of the forward force is about how flaws and incidents of unethical behavior in the organization are disapproved of primarily due to the growing expectation that the organization should maintain and not allow to decline its commendable ethical level. After all, an organization is only really ethical if it can maintain its level of ethics. Because the future provides evidence for the current level of ethics, the mantra of this effect is “Good should remain perfect.” Subsequently, when they become more ethical, organizations may experience having less room to disclose unethical behavior and will then be less likely to inform their stakeholders when unethical behavior occurs. This means that as the ethical level of an organization increases, so do the pressure and tendency for the organization to maintain its ethical reputation and keep up appearances.

Research shows elements of the keeping-up appearances effect. Asgari et al. (2021) report that high performing employees may face higher expectations from their stakeholders to stay at this level, which may lead to these stars becoming more self-interested. Star employees also struggle more with failures than less talented ones do, and any disruption in their performance is also more harshly evaluated. Baer et al. (2015) find that bus drivers who feel trusted increased their perceived workload and their concerns about reputation maintenance intensified, which in turn increased their exhaustion and burdened their performance. Koehler and Gershoff (2003) found that people respond more negatively and recommend more severe punishments when a trusted person or institution violates that trust. The scandals exposed by the 2017 #MeToo movement against sexual violence and intimidation had especially negative consequences for accused high-status individuals (Dewan & Jensen, 2020). Many other studies also show that people with a more honorable reputation are judged more harshly for their crimes, transgressions, and other defects (e.g., Kakkar et al., 2020; Rosoff, 1989; Skolnick & Shaw, 1994). Other studies show that the same holds for organizations. Based on an analysis of more than 500 employment discrimination suits, McDonnell and King (2018) find that the prestige of a company is positively related to the severity of punishments for transgressions. Dewan and Jensen (2020) find that the status of organizations increases the likelihood of the U.S. Securities and Exchange Commission’s enforcement action when the misconduct is part of a multiple-actor scandal. McDonnell et al. (2015) conclude that organizations that have greater reputational standing are more likely to be targeted by activist groups when they fail to live up to expected behavioral standards.

Social psychological theories of status are useful in explaining the keeping-up appearances effect. McDonnell and King (2018) use status characteristics theory and expectations states theory to explain why organizations with status and reputation are punished more severely in employment discrimination suits. Prestige influences audience evaluations by shaping expectations: having a good reputation for behaving a certain way will lead audience to expect positive behavior in that domain; while a bad reputation will have the opposite effect on expectations. Consequently, once an organization has been found guilty of a transgression, evaluators will punish organizations with high reputations more harshly than they will those with low reputations. McDonnell and King call this the halo tax. Instead of working as a buffer when an organization is accused of unethical behavior (the Matthew effect), a positive reputation will more likely attract harsher punishments than a less prestigious one will because the evaluators may feel especially duped, jilted, or betrayed when a trusted and admired organization deviates. Fragale et al. (2009) also find that evaluators are more likely to punish high-status wrongdoers because the latter are perceived to be more intentional and thus more deserving of harsh punishments than lower-status transgressors. McDonnell and King’s interpretation of status theory can be applied to the ethical development of organizations. The more the stakeholders consider an organization to be ethical—i.e., the better its ethical reputation (Ramakrishna Velamuri et al., 2017)—the higher are the stakeholders’ expectations that the organization stays on that ethical level, and the stronger the stakeholders’ disapproval when they learn about unethical behavior in and by the organization.

The keeping-up appearances effect may lead to unethical behavior when defects are hidden to maintain the ethical reputation of the organization. When stakeholders’ disapproval of the unethical behavior becomes stronger, people within the organization may be less likely to report such incidents because the consequences of reporting may be harsher. This makes impression management, like window dressing or ethics washing, and decoupling more likely. Not informing stakeholders about unethical behavior is unethical when stakeholders have the right to be informed about it (Van Oosterhout et al., 2006). When it is accepted within organizations that the higher the ethical reputation of an organization, the more the organization will be trusted when the accusations are not established, then it becomes more likely for organizations with a higher (rather than lower) ethical reputation to ignore and even lie about accusations (cf. Cole & Chandler, 2019). When not informing stakeholders leads to cover-ups, the situation then worsens and becomes even destructive because cover-ups mean more unethical behaviors, like fraud, destroying documents, silencing those involved, and obstructing whistleblowers (Ashforth & Anand, 2003). When stakeholders learn about these cover-ups, their disapproval will be even stronger because the scale of unethical behavior is bigger—what is being covered up and the cover-up itself—and the bad intentions are more evident (cf. Kundro & Nurmohamed, 2021). The disapproval of stakeholders becomes even stronger when it appears that incident concealment and dishonesty has become the new norm in the organization (Leavitt & Sluss, 2015) and that this new norm leads to the further escalation (Zyglidopoulos et al., 2009) and spirals of unethical behavior (Den Nieuwenboer & Kaptein, 2008). It would be even more difficult for organizations to disclose their defects. As a result, unethical behaviors may be hidden until due to their magnitude either the organization has no other option but to report them externally or others detect them. The paradox is the ethical reputation that was established by becoming more ethical increases the risks of unethical behavior just to maintain that ethical reputation. Where reputation is seen as having a social control function—organizations avoid unethical behavior to prevent reputational damage (Bednar et al., 2015)—the reputation itself may also lead to unethical behavior.

#### P8

The more ethical an organization becomes, the more it aims to maintain its ethical reputation, thus making unethical behavior by employees more likely.

## Discussion

**Fig. 2:**
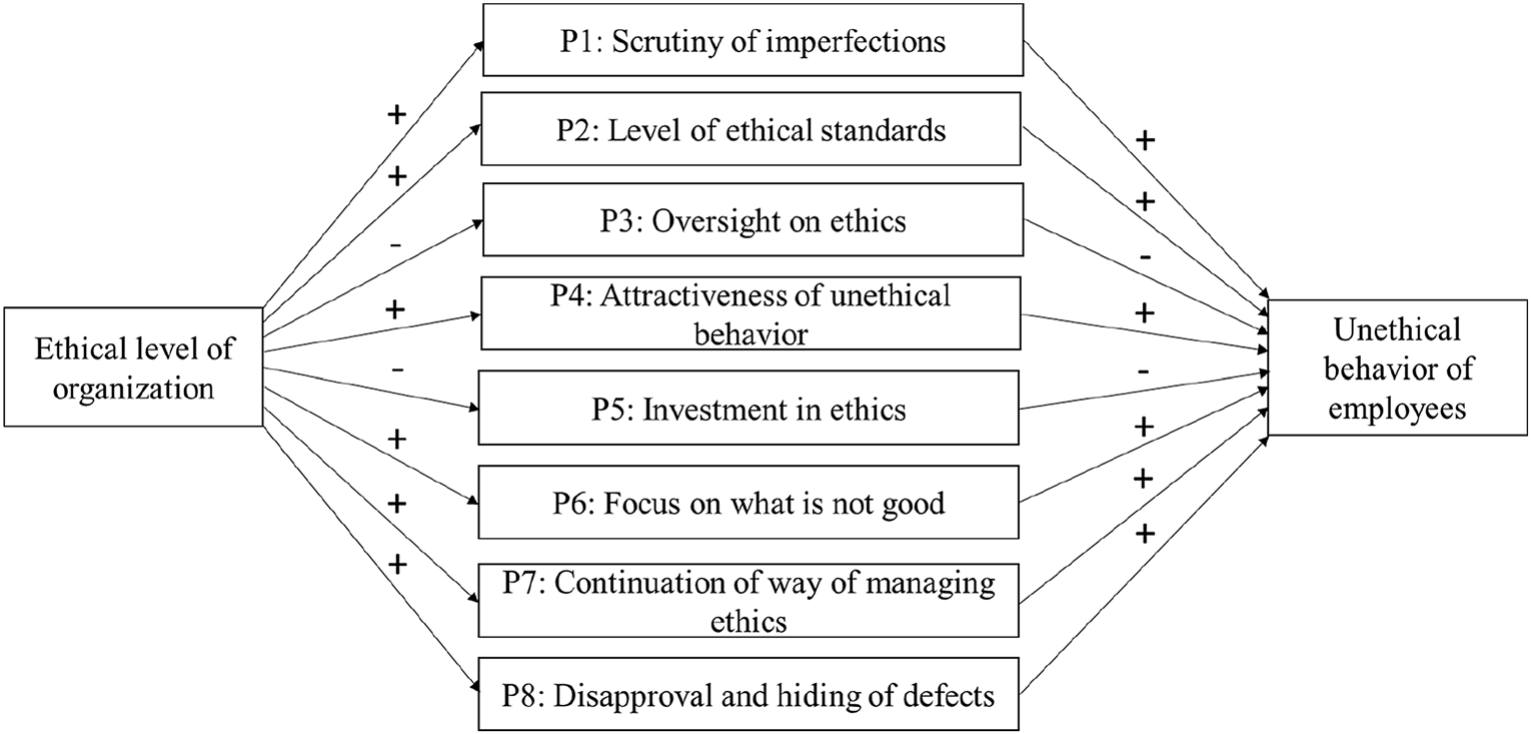
Overview of propositions

This article followed a new approach in explaining unethical behavior in organizations: by exploring whether unethical behavior can be caused by organizations becoming or being ethical. This article used the good barrel approach instead of the bad barrel approach and identified four threatening forces on the ethical organization. Each of these four forces was illustrated using two effects, making eight effects in total. Studies were presented that supported the practical existence of these effects. Each effect could be explained by a specific theory; and an explanation could be given for how each effect could lead to unethical behavior in organizations, producing the eight propositions shown in Fig. 2. In this manner, this article introduced a paradox of ethics, where goodness breeds badness and organizations that become more ethical face, in some respects, a higher likelihood of unethical behavior.

### Research Directions

The article makes possible at least seven research directions. These directions also address the limitations of the article.

A first research direction concerns the existence of the identified forces and effects and their impact on unethical behavior. This article tried to conceptually identify threatening forces and effects on the ethical organization, and although studies were found that indicate their existence, empirical research is needed to validate their existence. Many new studies on the existence of each of the mentioned effects within organizations in different sectors and countries are possible. When evidence for the existence of the forces and effects are found, then it is feasible to examine whether, as expressed in the eight propositions of this article, these forces and effects indeed increase the likelihood of unethical behavior in organizations. In this regard, it is relevant to examine not only the individual impact on unethical behavior of each effect but also their aggregated impact when an organization is confronted with multiple effects. When the effects and their impact on unethical behavior have been established, it is relevant to identify the circumstances that trigger the effects. For example, larger organizations may be more prone to the effects than smaller organizations are (cf. Durand et al., 2019). Thought-provoking research can also be done on the circumstances that determine whether an effect has a positive or a negative impact on unethical behavior. For example, the introduction of new standards (the high-jump bar effect) and the active inertia of ethics management (the repeat-prescription effect) may also have negative impact on unethical behavior when the new standards are met and when inertia leads to stable and reliable ethics management. It would also be interesting to examine what the net impact is on unethical behavior of organizations becoming more ethical when the threatening effects increase the likelihood of unethical behavior and the likelihood of unethical behavior decreases due to the reduction of the factors that make the organization a bad barrel.

A second research direction concerns expanding the explanation for the identified effects. In this article, one theory and one explanation for each effect’s impact on unethical behavior was presented. It would then be useful to explore whether there are other theories that can better explain the effects and whether there are other explanations for the impact of the effects on unethical behavior. For instance, the forbidden-fruit effect may also be explained by the concept of moral licensing: the more good deeds an organization does, the more the people within the organization start to believe that they have the license, the right, to do something bad (Blanken et al., 2015; Merritt et al., 2010). Another example is the Expectancy Theory of Vroom (1964) possibly explaining the gold digger effect. This effect may also lead to organizations overreacting in their ethics management when they do too much of a good thing—for instance, setting too many and too detailed standards, committing employees too much to ethics, and becoming too transparent about their ethics—which increases the likelihood of unethical behavior, as Kaptein argues (2017).

A third research direction opened up by this article is research into the relationships among the threatening effects. Although the article described the effects independently, this does not imply that there can be no relationship among the effects. Empirical research has to sort out how the effects are related to each other. It is possible that the effects exclude one another. For example, the gold digger effect is about closer inspection, while the retreating-cat effect is about less inspection, and the moving-spotlight effect is about changing the topic of the inspection. However, effects can still coexist in an organization when, for example, stakeholders respond differently to their organization becoming more ethical: some stakeholders inspect closer, while others inspect less or differently. Another possibility is that the effects may positively influence each other. For example, raising standards (the high-jump bar effect) can trigger the moving-spotlight effect when an organization focuses only on these higher standards. The keeping-up appearances effect may be triggered when the other effects lead to unethical behavior that should be hidden. The combination of effects may also reinforce the impact on unethical behavior. For instance, when the standards are raised (the high-jump bar effect) and the investment in ethics is reduced (the cheese slicer effect) simultaneously, the risk of unethical behavior grows because the expectations increase and the efforts decrease. Research on the relationships among the effects may also lead to identifying configurations of effects, where the effects exist in different sets of relations. Similar research could be conducted on the mutual relationships among the four identified forces.

A fourth research direction concerns the existence of other forces and effects. Although no less than four forces and eight effects were identified, this article does not claim to have given a complete overview of the threatening forces and effects on the ethical organization. As the identified forces seem logical and the identified effects plausible, conceptual and empirical research could identify other such threatening forces and effects. There could be inductive research among organizations that become or are ethical on whether there are indeed factors related to the goodness of the organization and which stimulate unethical behavior. Such research can provide evidence for the identified effects and forces and also for those that have not been identified here. Other research may examine how the effects identified in this article relate to other effects identified in the business ethics literature, such as the rebound effect (Berkhout et al., 2000; Sorrell & Dimitropoulos, 2008), the boomerang effect (Schlosser et al., 2012), the perverse effect (Cain et al., 2005), and the countervailing effect (Gao et al., 2012).

A fifth research direction concerns the further development and application of the good barrel approach. One of the contributions of the current article is this new perspective of the good barrel. This perspective was used to identify and discuss the threatening factors that are triggered when the organization is or becomes ethical. However, much more research can be done using the good barrel approach to explain unethical behavior. While there have been studies on how unethical goals lead to unethical behavior (Ordóñez & Welsh, 2015), the good barrel approach is about examining how ethical goals can also lead to unethical behavior. There have been studies on how a lack of monitoring leads to the failure to detect unethical behavior (Vaughan, 1996) and even to unethical behavior (Meyer & Rowan, 1977), but the good barrel approach is about studying how monitoring of unethical behavior can lead to unethical behavior. This approach can also enrich theories because the explanation for unethical behavior is not only the bad organization but also the good organization. For example, institutional theory explains decoupling by organizations as due to their lack of integrity and abilities (Boxenbaum & Jonsson, 2017); the good barrel approach is about organizations that have to decouple due to their integrity and ability. In this regard, more conceptual research is needed on what the good barrel entails, in the sense of what the ethical development of an organization contains. This article used current operationalizations of these concepts (Kaptein, 1998, 2008; Reidenbach & Robin, 1991; Victor & Cullen, 1988), but more research is needed to better define when an organization becomes more ethical so that there can be deeper and better understanding of when exactly an organization becomes more ethical and how this triggers the threatening forces and effects. In operationalizing the ethical content of organizations, it is interesting to examine whether the ethical content also entails the ability to withstand the threatening forces and effects and whether current conceptions of the ethical organization as a bad barrel should be enriched with the good barrel conception.

A sixth research direction concerns studying the dynamics of the ethical development of organizations. Reidenbach and Robin (1991, p. 284) concluded that their article “represents a start in the study of the dynamics of corporate moral development.” The model proposed above presents a new lens for studying the causes of the slowdown, stagnation, and even the decline of the ethical development of organizations. Similar to what has been done in the broader field of organizational life cycles (Mosca et al., 2021), future research may establish the possible life cycles in the ethical development of organizations. For example, we may expect that the ethical development of an organization goes up and down alternatingly. When the ethical development declines due to threatening forces and their effects, these threatening forces and their effects will increasingly become weaker thereby increasingly slowing down the decline of the ethical development of the organization. Subsequently when the organization starts to improve in its ethical development, the cycle may start all over again.

A final research direction is on the development of effective strategies to deal with threatening forces and effects. In the current article, the forces and effects are explained as being triggered when organizations become more ethical, and they are based on beliefs that are supposedly present within and around the organization. Research could be initiated on how these beliefs could be changed. How the impact of the effects on unethical behavior can be reduced or even canceled is another possible research. Given that the perspective of the good barrel approach is new in the field of ethics management, innovation in ethics management (Haidt & Treviño, 2017; Hogenbirk & Van Dun, 2021) may be needed to develop specific interventions and instruments.

### Practical Implications

The practical implication of the approach and model presented here is the importance for managers of reducing not only the “bad barrel” factors that explain unethical behavior but also the factors that are triggered when the organization is or becomes more ethical. The forces and effects that have been identified here suggest that developing and safeguarding the ethics of an organization becomes more difficult and less effective when the organization becomes more ethical. To manage the ethics of organizations effectively, the forces and effects should be acknowledged and understood. This means that those who manage the ethics of their organization should know which of the forces and effects are present and how these evolve when the organization becomes more ethical.

When threatening forces and effects are present and become stronger, those who manage the ethics of their organization should define how to best deal with these forces and effects if these are to be prevented from leading to unethical behavior. These managers can try to change the beliefs that shape the forces and the effects. For example, they can try to convince those who set the standards for the organization that becoming or being an ethical organization does not necessarily imply that new responsibilities should always be added (upward force), or to convince those who evaluate the organization that becoming or being an ethical organization does not necessarily mean that there will be no future defects in the organization (forward force). This article presented arguments to help convince those who create the effects that their actions, even if their motive is to reduce unethical behavior, can tragically lead to more unethical behavior. However, when the beliefs cannot be changed sufficiently, managers can try to prevent the effects from leading to unethical behavior by mitigating the effects. For example, when external supervisors retreat, organizations can increase their own oversight in general, and more specifically, regarding the “forbidden-fruit” that is becoming more attractive (the downward force); or organizations can set an absolute minimum level of investments in ethics management (the backward force). When the threatening forces and effects are successfully addressed, fortunately the ethical paradox discussed here will not become a reality.

## References

[CR1] Acemoglu, D., & Wolitzky, A. (2012). Cycles of distrust: An economic model. *Working Paper No. 18257*. National Bureau of Economic Research.

[CR2] Aguilera RV, Desender K, Bednar MK, Lee JH (2015). Connecting the dots: Bringing external corporate governance into the corporate governance puzzle. Academy of Management Annals.

[CR3] Albert S, Whetten DA, Cummings LL, Staw BM (1985). Organizational identity. Research in organizational behavior.

[CR202] Allen C, Imrie R (2016). The knowledge business: The commodification of urban and housing research.

[CR4] Al-Taie M, Cater-Steel A (2020). The organisational life cycle scale: An empirical validation. The Journal of Entrepreneurship.

[CR5] Andarge T, Lichtenberg E (2020). Regulatory compliance under enforcement gaps. Journal of Regulatory Economics.

[CR6] Armitage CJ, Wright CL, Parfitt G, Pegington M, Donnelly LS, Harvie MN (2014). Self-efficacy for temptations is a better predictor of weight loss than motivation and global self-efficacy: Evidence from two prospective studies among overweight/obese women at high risk of breast cancer. Patient Education and Counseling.

[CR7] Asgari E, Hunt RA, Lerner DA, Townsend DM, Hayward ML, Kiefer K (2021). Red giants or black holes? The antecedent conditions and multilevel impacts of star performers. Academy of Management Annals.

[CR8] Ashforth BE, Anand V (2003). The normalization of corruption in organizations. Research in Organizational Behavior.

[CR9] Ashforth BE, Mael F (1989). Social identity theory and the organization. Academy of Management Review.

[CR10] Ashforth BE, Schinoff BS (2016). Identity under construction: How individuals come to define themselves in organizations. Annual Review of Organizational Psychology and Organizational Behavior.

[CR11] Ashkanasy NM, Windsor CA, Treviño LK (2006). Bad apples in bad barrels revisited: Cognitive moral development, just world beliefs, rewards, and ethical decision-making. Business Ethics Quarterly.

[CR12] Audia PG, Locke EA, Smith KG (2000). The paradox of success: An archival and a laboratory study of strategic persistence following radical environmental change. Academy of Management Journal.

[CR13] Bachram H (2004). Climate fraud and carbon colonialism: The new trade in greenhouse gases. Capitalism Nature Socialism.

[CR14] Baer MD, Dhensa-Kahlon RK, Colquitt JA, Rodell JB, Outlaw R, Long DM (2015). Uneasy lies the head that bears the trust: The effects of feeling trusted on emotional exhaustion. Academy of Management Journal.

[CR15] Baldwin R, Black J (2016). Driving priorities in risk-based regulation: What’s the problem?. Journal of Law and Society.

[CR16] Bapuji H, Patel C, Ertug G, Allen DG (2020). Corona crisis and inequality: Why management research needs a societal turn. Journal of Management.

[CR17] Baumeister RF, Bratslavsky E, Finkenauer C, Vohs KD (2001). Bad is stronger than good. Review of General Psychology.

[CR18] Bednar MK, Love EG, Kraatz M (2015). Paying the price? The impact of controversial governance practices on managerial reputation. Academy of Management Journal.

[CR19] Belschak FD, Den Hartog DN (2009). Consequences of positive and negative feedback: The impact on emotions and extra-role behaviors. Applied Psychology.

[CR20] Bennett VM, Pierce L, Snyder JA, Toffel MW (2013). Customer-driven misconduct: How competition corrupts business practices. Management Science.

[CR21] Berkhout PH, Muskens JC, Velthuijsen JW (2000). Defining the rebound effect. Energy Policy.

[CR22] Berti M, Simpson AV (2021). The dark side of organizational paradoxes: The dynamics of disempowerment. Academy of Management Review.

[CR23] Binder A, Naderer B, Matthes J (2020). A “forbidden fruit effect”: An eye-tracking study on children’s visual attention to food marketing. International Journal of Environmental Research and Public Health.

[CR24] Blanken I, Van de Ven N, Zeelenberg M (2015). A meta-analytic review of moral licensing. Personality and Social Psychology Bulletin.

[CR25] Boulding KE (1950). A reconstruction of economics.

[CR26] Boxenbaum E, Jonsson S (2017). Isomorphism, diffusion and decoupling: Concept evolution and theoretical challenges. The Sage Handbook of Organizational Institutionalism.

[CR27] Braithwaite J, Braithwaite V, Levi M (1998). Institutionalizing distrust, enculturating trust. Trust and Governance.

[CR28] Braithwaite J (2002). Rewards and regulation. Journal of Law and Society.

[CR29] Braithwaite V (2007). Responsive regulation and taxation: Introduction. Law & Policy.

[CR30] Braithwaite J (2020). Regulatory mix, collective efficacy, and crimes of the powerful. Journal of White Collar and Corporate Crime.

[CR31] Brehm JW (1966). A theory of psychological reactance.

[CR32] Briscoe F, Gupta A, Anner MS (2015). Social activism and practice diffusion: How activist tactics affect non-targeted organizations. Administrative Science Quarterly.

[CR33] Bushman BJ, Stack AD (1996). Forbidden fruit versus tainted fruit: Effects of warning labels on attraction to television violence. Journal of Experimental Psychology: Applied.

[CR34] Cain DM, Loewenstein G, Moore DA (2005). The dirt on coming clean: Perverse effects of disclosing conflicts of interest. The Journal of Legal Studies.

[CR35] Carroll AB (2021). Corporate social responsibility: Perspectives on the CSR construct’s development and future. Business & Society.

[CR36] Clark CE, Newell S (2013). Institutional work and complicit decoupling across the US capital markets: The work of rating agencies. Business Ethics Quarterly.

[CR37] Clarke RV (2012). Opportunity makes the thief. Really? And so what?. Crime Science.

[CR38] Cole BM, Chandler D (2019). A model of competitive impression management: Edison versus Westinghouse in the war of the currents. Administrative Science Quarterly.

[CR39] Colquitt JA, Rodell JB (2011). Justice, trust, and trustworthiness: A longitudinal analysis integrating three theoretical perspectives. Academy of Management Journal.

[CR40] Consumer Reports National Research Center (2011). Survey: Consumers are running risks by postponing car maintenance or repair. *Consumer Reports News*: December 20.

[CR41] De Jonge B, Dijkstra AS, Romeijnders W (2015). Cost benefits of postponing time-based maintenance under lifetime distribution uncertainty. Reliability Engineering & System Safety.

[CR42] Den Nieuwenboer NA, Kaptein M (2008). Spiraling down into corruption: A dynamic analysis of the social identity processes that cause corruption in organizations to grow. Journal of Business Ethics.

[CR43] Dewan Y, Jensen M (2020). Catching the big fish: The role of scandals in making status a liability. Academy of Management Journal.

[CR44] Dilger, A. (2002). Never change a winning Team: An analysis of hazard rates in the NBA. *Greifswald Economics Working Paper No. 3/02*. Retrieved Oct 29, 2019 from https://ssrn.com/abstract=312001

[CR45] Durand R, Hawn O, Ioannou I (2019). Willing and able: A general model of organizational responses to normative pressures. Academy of Management Review.

[CR46] Dutton JE, Ashford SJ (1993). Selling issues to top management. Academy of Management Review.

[CR47] Eberl P, Geiger D, Aßländer MS (2015). Repairing trust in an organization after integrity violations: The ambivalence of organizational rule adjustments. Organization Studies.

[CR48] Eberlein B (2019). Who fills the global governance gap? Rethinking the roles of business and government in global governance. Organization Studies.

[CR49] Ethics & Compliance Initiative (2021). 2021 Global business ethics survey report: The state of ethics & compliance in the workplace.

[CR50] Felson, M., & Clarke, R. V. (1998). Opportunity makes the thief. *Police Research Series. Paper**98.*

[CR51] Ferrin DL, Bligh MC, Kohles JC (2007). Can I trust you to trust me? A theory of trust, monitoring, and cooperation in interpersonal and intergroup relationships. Group & Organization Management.

[CR52] FitzGibbon, L., Ogulmus, C., Fastrich, G. M., Lau, J. K. L., Aslan, S., Lepore, L., & Murayama, K. (2020). Understanding the forbidden fruit effect: People's desire to see what is forbidden and unavailable. OSF Preprints. 10.31219/osf.io/ndpw

[CR53] Flammer C (2013). Corporate social responsibility and shareholder reaction: The environmental awareness of investors. Academy of Management Journal.

[CR54] Ford C, Hess D (2008). Can corporate monitorships improve corporate compliance. Journal of Corporation Law.

[CR55] Fragale AR, Rosen B, Xu C, Merideth I (2009). The higher they are, the harder they fall: The effects of wrongdoer status on observer punishment recommendations and intentionality attributions. Organizational Behavior and Human Decision Processes.

[CR56] Gao TT, Leichter G, Wei YS (2012). Countervailing effects of value and risk perceptions in manufacturers’ adoption of expensive, discontinuous innovations. Industrial Marketing Management.

[CR57] Gelter M, Helleringer G (2018). Opportunity makes a thief: Corporate opportunities as legal transplant and convergence in corporate law. Berkeley Business Law Journal.

[CR58] Gilovich T, Savitsky K (1999). The spotlight effect and the illusion of transparency: Egocentric assessments of how we are seen by others. Current Directions in Psychological Science.

[CR59] Gino F, Schweitzer ME, Mead NL, Ariely D (2011). Unable to resist temptation: How self-control depletion promotes unethical behavior. Organizational Behavior and Human Decision Processes.

[CR60] Gosselt JF, Van Hoof JJ, De Jong MD (2012). Why should i comply? Sellers' accounts for (non-) compliance with legal age limits for alcohol sales. Substance Abuse Treatment, Prevention, and Policy.

[CR61] Graafland JJ (2002). Profits and principles: Four perspectives. Journal of Business Ethics.

[CR62] Greenwood DJ (2004). Enronitis: Why good corporations go bad. Columbia Business. Law Review.

[CR63] Gupta YP, Chin DC (1994). Organizational life cycle: A review and proposed directions for research. Mid-Atlantic Journal of Business.

[CR64] Haack P, Martignoni D, Schoeneborn D (2021). A bait-and-switch model of corporate social responsibility. Academy of Management Review.

[CR65] Hahn T, Pinkse J, Preuss L, Figge F (2016). Ambidexterity for corporate social performance. Organization Studies.

[CR66] Haidt J, Treviño L (2017). Make business ethics a cumulative science. Nature Human Behaviour.

[CR67] Hamid MY, Alexander K, Baldry D (2007). The cause and effects of deferred maintenance on higher education buildings. Journal of the University of Salford.

[CR68] Heine K, Rindfleisch H (2013). Organizational decline: A synthesis of insights from organizational ecology, path dependence and the resource-based view. Journal of Organizational Change Management.

[CR69] Hoekstra A, Kaptein M (2021). The integrity of integrity programs: Toward a normative framework. Public Integrity.

[CR70] Hogenbirk S, Van Dun DH (2021). Innovative ethics officers as drivers of effective ethics programs: An empirical study in the Netherlands. Business Ethics: THe Environment &amp; Responsibility.

[CR71] Hoogenboezem JA, Hoogenboezem DB (2005). Coping with targets: Performance measurement in The Netherlands police. International Journal of Productivity and Performance Management.

[CR72] Institute for Business Ethics (2021). Ethics at work: Survey of employees.

[CR73] Iso-Ahola SE, Dotson CO (2014). Psychological momentum: Why success breeds success. Review of General Psychology.

[CR75] Jawahar IM, McLaughlin GL (2001). Toward a descriptive stakeholder theory: An organizational life cycle approach. Academy of Management Review.

[CR76] Jones TM (1991). Ethical decision making by individuals in organizations: An issue-contingent model. Academy of Management Review.

[CR77] Jones TM (1995). Instrumental stakeholder theory: A synthesis of ethics and economics. Academy of Management Review.

[CR78] Jones GR, George JM (1998). The experience and evolution of trust: Implications for cooperation and teamwork. Academy of Management Review.

[CR79] Jones KL, Tymms P, Kemethofer D, O’Hara J, McNamara G, Huber S, Myrberg E, Skedsmo G, Greger D (2017). The unintended consequences of school inspection: The prevalence of inspection side-effects in Austria, the Czech Republic, England, Ireland, the Netherlands, Sweden, and Switzerland. Oxford Review of Education.

[CR80] Kahn WA (1990). Toward an agenda for business ethics research. Academy of Management Review.

[CR81] Kakkar H, Sivanathan N, Gobel MS (2020). Fall from grace: The role of dominance and prestige in the punishment of high-status actors. Academy of Management Journal.

[CR201] Kaptein M (1998). Ethics management: Auditing and developing the ethical content of organizations.

[CR82] Kaptein M (2008). Developing and testing a measure for the ethical culture of organizations: The corporate ethical virtues model. Journal of Organizational Behavior.

[CR83] Kaptein M (2011). Understanding unethical behavior by unraveling ethical culture. Human Relations.

[CR84] Kaptein M (2017). When organizations are too good: Applying Aristotle's doctrine of the mean to the corporate ethical virtues model. Business Ethics: A European Review.

[CR85] Kaptein M (2019). The moral entrepreneur: A new component of ethical leadership. Journal of Business Ethics.

[CR86] Kaptein, M. (2021). Issue-driven progress in business ethics: When the responsibility to protect values requires companies to introduce new norms. 10.31124/advance.14983848.v1

[CR87] Kaptein M, Wempe JFDB (2002). The balanced company: A theory of corporate integrity.

[CR88] Kassin SM, Goldstein CC, Savitsky K (2003). Behavioral confirmation in the interrogation room: On the dangers of presuming guilt. Law and Human Behavior.

[CR89] Kerr S (1975). On the folly of rewarding A, while hoping for B. Academy of Management Journal.

[CR90] Kish-Gephart JJ, Harrison DA, Treviño LK (2010). Bad apples, bad cases, and bad barrels: Meta-analytic evidence about sources of unethical decisions at work. Journal of Applied Psychology.

[CR91] Koehler JJ, Gershoff AD (2003). Betrayal aversion: When agents of protection become agents of harm. Organizational Behavior and Human Decision Processes.

[CR92] Koo M, Fishbach A (2010). Climbing the goal ladder: How upcoming actions increase level of aspiration. Journal of Personality and Social Psychology.

[CR93] Kranz AM, Gahlon G, Dick AW, Stein BD (2021). Characteristics of US adults delaying dental care due to the COVID-19 pandemic. JDR Clinical & Translational Research.

[CR94] Kundro TG, Nurmohamed S (2021). Understanding when and why cover-ups are punished less severely. Academy of Management Journal.

[CR95] Lazear EP (2004). The Peter principle: A theory of decline. Journal of Political Economy.

[CR96] Leavitt K, Sluss DM (2015). Lying for who we are: An identity-based model of workplace dishonesty. Academy of Management Review.

[CR97] Lehman DW, Ramanujam R (2009). Selectivity in organizational rule violations. Academy of Management Review.

[CR98] Lewin K (1951). Field theory in social sciences.

[CR100] Leonidou LC, Aykol B, Fotiadis TA, Christodoulides P, Zeriti A (2017). Betrayal in international buyer-seller relationships: Its drivers and performance implications. Journal of World Business.

[CR101] Locke EA, Latham GP (2019). The development of goal setting theory: A half century retrospective. Motivation Science.

[CR102] Lynn AP (2020). Why ‘doing well by doing good’ goes wrong: A critical review of good ethics pays’ claims in managerial thinking. Academy of Management Review.

[CR103] Mahon JF, Waddock SA (1992). Strategic issues management: An integration of issue life cycle perspectives. Business & Society.

[CR104] Mann T, Ward A (2001). Forbidden fruit: Does thinking about a prohibited food lead to its consumption?. International Journal of Eating Disorders.

[CR105] Marshall, A. (1890/2009). *Principles of economics*. Cosimo.

[CR106] Martinez-Moyano IJ, McCaffrey DP, Oliva R (2014). Drift and adjustment in organizational rule compliance: Explaining the “regulatory pendulum” in financial markets. Organization Science.

[CR107] Mayer C (2021). The future of the corporation and the economics of purpose. Journal of Management Studies.

[CR108] Mayer RC, Davis JH, Schoorman FD (1995). An integrative model of organizational trust. Academy of Management Review.

[CR109] McDonnell MH, King BG (2018). Order in the court: How firm status and reputation shape the outcomes of employment discrimination suits. American Sociological Review.

[CR110] McDonnell MH, King BG, Soule SA (2015). A dynamic process model of private politics: Activist targeting and corporate receptivity to social challenges. American Sociological Review.

[CR111] McKinley W, Latham S, Braun M (2014). Organizational decline and innovation: Turnarounds and downward spirals. Academy of Management Review.

[CR112] McMillan JJ, White RA (1993). Auditors’ belief revisions and evidence search: The effect of hypothesis frame, confirmation bias, and professional skepticism. Accounting Review.

[CR113] Meister A, Jehn KA, Thatcher SM (2014). Feeling misidentified: The consequences of internal identity asymmetries for individuals at work. Academy of Management Review.

[CR114] Merritt AC, Effron DA, Monin B (2010). Moral self-licensing: When being good frees us to be bad. Social and Personality Psychology Compass.

[CR115] Methot JR, Lepak D, Shipp AJ, Boswell WR (2017). Good citizen interrupted: Calibrating a temporal theory of citizenship behavior. Academy of Management Review.

[CR116] Meyer JW, Rowan B (1977). Institutionalized organizations: Formal structure as myth and ceremony. American Journal of Sociology.

[CR117] Miller D (1992). The Icarus paradox: How exceptional companies bring about their own downfall. Business Horizons.

[CR118] Mishina Y, Dykes BJ, Block ES, Pollock TG (2010). Why “good” firms do bad things: The effects of high aspirations, high expectations, and prominence on the incidence of corporate illegality. Academy of Management Journal.

[CR119] Moody-Adams MM (1999). The idea of moral progress. Metaphilosophy.

[CR120] Mosca L, Gianecchini M, Campagnolo D (2021). Organizational life cycle models: A design perspective. Journal of Organization Design.

[CR121] Ocasio W (1997). Towards an attention-based view of the firm. Strategic Management Journal.

[CR122] Ocasio W (2011). Attention to attention. Organization Science.

[CR123] Olekalns M, Caza BB, Vogus TJ (2020). Gradual drifts, abrupt shocks: From relationship fractures to relational resilience. Academy of Management Annals.

[CR124] Ordóñez LD, Welsh DT (2015). Immoral goals: How goal setting may lead to unethical behavior. Current Opinion in Psychology.

[CR125] Ozcan P, Gurses K (2018). Playing cat and mouse: Contests over regulatory categorization of dietary supplements in the United States. Academy of Management Journal.

[CR126] Peter LJ, Hull R (1969). The Peter principle: Why things always go wrong.

[CR127] Porter ME (1980). Competitive strategy: Techniques for analyzing industries and competitors.

[CR128] Putnam L, Thayer L (1986). Contradictions and paradoxes in organizations. Organization communications: Emerging perspectives.

[CR129] Quintelier A, Vanhoof J, De Maeyer S (2018). Understanding the influence of teachers’ cognitive and affective responses upon school inspection feedback acceptance. Educational Assessment, Evaluation and Accountability.

[CR130] Ramakrishna Velamuri S, Venkataraman S, Harvey WS (2017). Seizing the ethical high ground: Ethical reputation building in corrupt environments. Journal of Management Studies.

[CR131] Reidenbach RE, Robin DP (1991). A conceptual model of corporate moral development. Journal of Business Ethics.

[CR132] Rose AM, Rose JM, Suh I, Thibodeau J, Linke K, Norman CS (2021). Why financial executives do bad things: The effects of the slippery slope and tone at the top on misreporting behavior. Journal of Business Ethics.

[CR133] Rosoff SM (1989). Physicians as criminal defendants: Specialty, sanctions, and status liability. Law and Human Behavior.

[CR134] Scandura TA, Gower K (2019). Management today: Best practices for the modern workplace.

[CR135] Schaubroeck JM, Peng AC, Hannah ST, Ma J, Cianci AM (2021). Struggling to meet the bar: Occupational progress failure and informal leadership behavior. Academy of Management Journal..

[CR136] Schlosser F, Zinni D, Armstrong-Stassen M (2012). Intention to unretire: HR and the boomerang effect. Career Development International.

[CR137] Schneider HS (2012). Agency problems and reputation in expert services: Evidence from auto repair. The Journal of Industrial Economics.

[CR138] Schwab B (1996). A note on ethics and strategy: Do good ethics always make for good business?. Strategic Management Journal.

[CR139] Short JL, Toffel MW (2010). Making self-regulation more than merely symbolic: The critical role of the legal environment. Administrative Science Quarterly.

[CR141] Singer P (1981). The expanding circle.

[CR140] Singh J, Svensson G, Wood G, Callaghan M (2011). A longitudinal and cross-cultural study of the contents of codes of ethics of Australian, Canadian and Swedish corporations. Business Ethics: A European Review.

[CR142] Sitkin SB, Miller CC, See KE (2017). The stretch goal paradox: Audacious targets are widely misunderstood and widely misused. Harvard Business Review.

[CR143] Skolnick P, Shaw JI (1994). Is defendant status a liability or a shield? Crime severity and professional relatedness. Journal of Applied Social Psychology.

[CR144] Smith JF, Kida T (1991). Heuristics and biases: Expertise and task realism in auditing. Psychological Bulletin.

[CR145] Smith WK, Lewis MW (2011). Toward a theory of paradox: A dynamic equilibrium model of organizing. Academy of Management Review.

[CR146] Smith KG, Mitchell TR, Summer CE (1985). Top level management priorities in different stages of the organizational life cycle. Academy of Management Journal.

[CR147] Soetens B, Braet C, Moens E (2008). Thought suppression in obese and non-obese restrained eaters: Piece of cake or forbidden fruit?. European Eating Disorders Review: THe Professional Journal of the Eating Disorders Association.

[CR148] Solinger ON, Jansen PG, Cornelissen JP (2020). The emergence of moral leadership. Academy of Management Review.

[CR149] Sorrell S, Dimitropoulos J (2008). The rebound effect: Microeconomic definitions, limitations and extensions. Ecological Economics.

[CR150] Stead WE, Worrell DL, Stead JG (1990). An integrative model for understanding and managing ethical behavior in business organizations. Journal of Business Ethics.

[CR151] Strickland LH (1958). Surveillance and trust. Journal of Personality.

[CR152] Sull DN (1999). The dynamics of standing still: Firestone Tire & Rubber and the radial revolution. Business History Review.

[CR153] Sull DN (2005). Why good companies go bad and how great managers remake them.

[CR154] Tanyi P, Litt B (2017). The unintended consequences of the frequency of PCAOB inspection. Journal of Business Finance & Accounting.

[CR155] Taylor B (2006). Shell shock: Why do good companies do bad things?. Corporate Governance: An International Review.

[CR157] Treviño LK, Youngblood SA (1990). Bad apples in bad barrels: A causal analysis of ethical decision-making behavior. Journal of Applied Psychology.

[CR158] Treviño LK, Den Nieuwenboer NA, Kish-Gephart JJ (2014). (Un)ethical behavior in organizations. Annual Review of Psychology.

[CR159] Van Oosterhout J, Heugens PP, Kaptein M (2006). The internal morality of contracting: Advancing the contractualist endeavor in business ethics. Academy of Management Review.

[CR200] Victor B, Cullen JB (1988). The organizational bases of ethical work climates. Administrative Science Quarterly.

[CR160] Vroom VH (1964). Work and motivation.

[CR161] Vaughan D (1996). The challenger launch decision: Risky technology, culture, and deviance at NASA.

[CR162] Whetten DA (1987). Organizational growth and decline processes. Annual Review of Sociology.

[CR163] Wickert C, Scherer AG, Spence LJ (2016). Walking and talking corporate social responsibility: Implications of firm size and organizational cost. Journal of Management Studies.

[CR164] Williams P, Khan MS, Naumann E (2011). Customer dissatisfaction and defection: The hidden costs of downsizing. Industrial Marketing Management.

[CR165] Zahra SA, Priem RL, Rasheed AA (2005). The antecedents and consequences of top management fraud. Journal of Management.

[CR166] Zyglidopoulos SC, Fleming PJ, Rothenberg S (2009). Rationalization, overcompensation and the escalation of corruption in organizations. Journal of Business Ethics.

